# Calix[4]arene Derivative for Iodine Capture and Effect on Leaching of Iodine through Packaging

**DOI:** 10.3390/molecules28041869

**Published:** 2023-02-16

**Authors:** Loredana Ferreri, Marco Rapisarda, Melania Leanza, Cristina Munzone, Nicola D’Antona, Grazia Maria Letizia Consoli, Paola Rizzarelli, Emanuela Teresa Agata Spina

**Affiliations:** 1 Institute of Biomolecular Chemistry, CNR, Via Paolo Gaifami 18, 95126 Catania, Italy; 2 Institute for Polymers, Composites and Biomaterials, CNR, Via Paolo Gaifami 18, 95126 Catania, Italy; 3 Department of Chemistry, University of Catania, Viale A. Doria 6, 95125 Catania, Italy

**Keywords:** iodine capture, calix[4]arene, povidone-iodine, polyethylene, polypropylene, iodophore, plastic packaging

## Abstract

A hydrophobic calix[4]arene derivative was investigated for its iodine (I_2_) capture efficiency from gaseous and liquid phase. The iodine uptake was followed by UV-vis spectroscopy. Additionally, the influence of the calix[4]arene derivative–polyolefin system on the leaching of iodine through packaging from a povidone-iodine-based (PVP-I) formulation was evaluated. In fact, iodine is a low-cost, multi-target, and broad-spectrum antiseptic. However, it is volatile, and the extended storage of I_2_-based formulations is challenging in plastic packaging. Here, we investigated the possibility of reducing the loss of I_2_ from an iodophor formulation by incorporating 4-*tert*-butylcalix [4]arene-tetraacetic acid tetraethyl ester (CX) and its iodine complex in high-density polyethylene (HDPE) or polypropylene (PP) via a swelling procedure. Surface and bulk changes were monitored by contact angle, thermogravimetric analysis (TGA), and UV-vis diffuse reflectance spectra. The barrier effect of the different polymeric systems (embedded with CX, iodine-CX complex, or I2) was evaluated by monitoring the I2 retention in a buffered PVP-I solution by UV-vis spectroscopy. Overall, experimental data showed the capability of the calix[4]arene derivative to complex iodine in solution and the solid state and a significant reduction in the iodine leaching by the PP-CX systems.

## 1. Introduction

Calix[n]arenes are a family of macrocyclic polyphenols characterized by synthetic versatility and the presence of an aromatic cavity that can host ions and neutral molecules [1]. The *p-tert*-butyl-calix[4]arene, the smallest oligomer of this family, can be blocked in a cone conformation by functionalization of the phenolic OH groups at the lower rim of the macrocycle [2]. The rigid ‘basket’ structure improves the possibilities of preparing host–guest or inclusion complexes [3]. The capability of the π-electron-rich cavity of the calix[4]arene macrocycle to complex iodine by halogen-π interactions has been reported. Calix[4]arene derivatives [4,5] and polymeric calixarenes [6,7,8] have provided functional compounds or nanosheets adsorbing iodine in vapor and solution phase with applications for pollutant removal [9,10,11].

Iodine has been known as an effective bactericide since 1800. However, its widespread use was limited by several undesirable factors, such as poor water solubility, limited chemical stability, and high local toxicity. Therefore, a new class of chemical complexes of iodine with organic polymers, known as iodophors, were investigated [12]. In particular, the complex of iodine with polyvinilpirrolidone (PVP-I) is largely utilized clinically due to its broad range of antimicrobial activity and unknown bacterial resistance. Povidone-iodine complex consists of PVP units that are linked with iodine via hydrogen bonds between two pyrroles and contains triiodide anions and a small amount of non-complexed mobile iodine (I_2_-free) that is the active bactericidal agent [13,14].

One of the main problems faced in the pharmaceutical formulations of PVP-I, as well as other organic iodophor solutions packaged in a plastic container for medical use, is the leaching of I_2_ through the packaging itself [15]. This involves both a decrease in stability and therapeutic capacity of the iodophor solutions. This problem mainly concerns low-concentration PVP-I solutions necessary for the treatment of delicate organs such as the eyes [15,16]. Indeed, the loss of iodine is a function of the packaging material for a given temperature and PVP-I concentration. In particular, the plastic containers in low-density polyethylene (LDPE) are more permeable to iodine than high-density polyethylene (HDPE) or polypropylene (PP) [17]. Glass (not permeable to iodine) represents the more suitable material for a container to store dilute PVP-I solutions. However, not even the glass containers can totally maintain, for a prolonged period, the stability of dilute solutions because of the plastic dropper, which may be an important source for the leaching of iodine.

To the best of our knowledge, calixarene derivatives have been employed as efficient catalysts for the polymerization of propylene [18,19] or stabilizers against PP oxidative degradation [20]. They have been grafted on PP [21] or mixed with PP [22], but never embedded in PP by the swelling method. In this study, we successfully investigated the capacity of a hydrophobic calix[4]arene derivative (tetra(ethoxycarbonyl-methoxy)-4-*tert*-butylcalix[4]arene, CX) to capture iodine from gaseous and liquid phase. Moreover, we explored the possibility of reducing the leaching of I_2_ through packaging from a PVP-I solution by embedding CX and its iodine complex in polymer matrices (HDPE or PP) via a swelling procedure. Comparative tests were carried out including polymer films embedded with I_2_ only. Surface and bulk changes were monitored by different analytical techniques (contact angle, TGA, UV). The barrier effect of the different polymeric systems (PP embedded with CX, iodine-loaded CX or I_2_) was evaluated by monitoring the iodine retention in a buffered PVP-I solution by UV-vis spectroscopy. The iodine complex of CX embedded in the PP films showed good stability over time and better reduced the leaching of iodine from the PVP-I buffered solution in comparison with unmodified polyolefin samples (barrier effect).

## 2. Results

### 2.1. Synthesis of Tetra(ethoxycarbonyl-methoxy)-4-tert-butylcalix[4]arene

**Figure 1:**
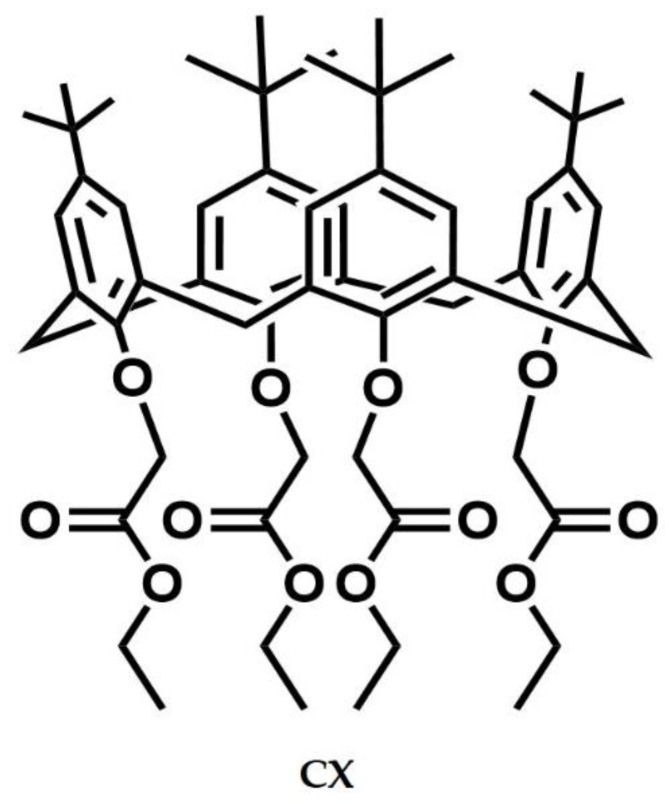
Molecular structure of the tetra(ethoxycarbonyl-methoxy)-4-*tert*-butylcalix[4]arene (CX).

tetra(ethoxycarbonyl-methoxy)-4-*tert*-butylcalix[4]arene (CX) was synthesized by following a procedure found in the literature [23]. In brief, the commercial 4-*tert*-butyl-calix[4]arene was treated with bromo-ethylacetate in the presence of potassium carbonate (molar ratio 1:10:10) in acetone as a solvent. CX was characterized by ^1^H NMR spectrum whose signals (Figure S1) were consistent with the structure depicted in Figure 1.

### 2.2. Formation of CX/Iodine Inclusion Complex

Iodine is one of the strongest electron acceptors. In principle, CX can host iodine in its π-electron-rich cavity and form a charge transfer complex. Accordingly, we explored the capability of CX to capture iodine in chloroform solution and in the solid state from iodine water solution (solid–liquid method) and iodine vapor (solid–air method).

#### 2.2.1. Formation of the Complex in Chloroform Solution

**Figure 2:**
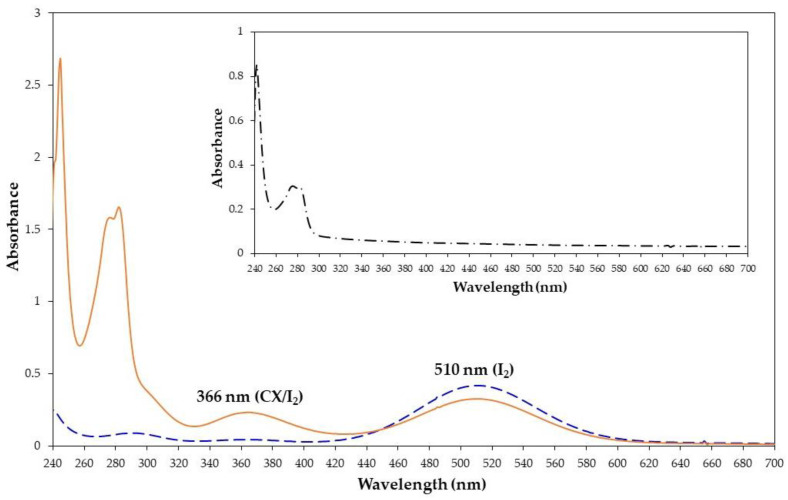
UV-vis spectra of iodine (1.3 × 10^−3^ M, dashed blue line), CX/iodine 1:1 molar ratio (solid orange line), and CX alone (inset) in chloroform at 25 °C.

To investigate the formation of a complex between CX and iodine, chloroform was chosen as an ideal solvent. The addition of molecular iodine to a chloroform solution of CX showed the iodine absorption band at 510 nm and the appearance of a new band at 366 nm (Figure 2) relative to the formation of the CX/iodine complex. This behavior was reported for other complexes of calix[4]arene derivatives with iodine [4,5]. The absorption at 366 nm was enhanced by increasing the molar excess of CX (from 1:0.5 to 1:3 molar ratio) (Figure S2).

Similar to other calix[4]arene derivatives, the binding stoichiometry of the CX/iodine complex was determined to be 1:1 and a log K of 3.95 was found for the complex formation constant (see ESI and Figures S3–S5).

#### 2.2.2. Formation of the Complex by Solid CX and Iodine in Water Solution (Solid–Liquid Method)

**Figure 3:**
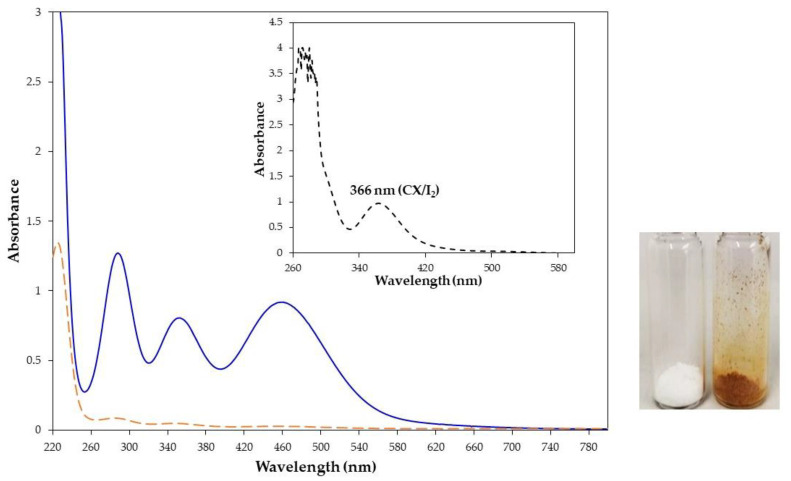
UV-vis spectra of iodine water solution (0.33 mg/mL, 1.3 × 10^−3^ M) before (solid blue line) and after stirring with CX (dashed orange line) and CX/iodine complex powder dissolved in chloroform (inset). Pictures of CX (white powder) and its complex with iodine after filtration (orange powder).

The water insoluble CX also showed the capability to capture iodine from an iodine water solution. When an excess (10:1 molar ratio) of CX (55 mg/mL) was added to a water solution of iodine (0.3 mg/mL), the white calixarene powder turned orange and the yellow water solution became discolored (Figure 3, inset). The capture of iodine by CX, visible by the naked eye, was corroborated by the comparison of the UV-vis spectra of the iodine water solution before and after the contact with CX (Figure 3). The absorbance of the iodine in water was reduced by 98% after the contact with CX. As further evidence of the complex formation, the UV-vis spectrum of the orange powder dissolved in chloroform showed the typical spectrum of the complex with absorption at 366 nm and no significant absorption at 510 nm relative to free iodine (Figure 3, inset).

The amount of iodine captured from the water solution depends on the CX amount, as evidenced by monitoring the reduction in the iodine absorption bands in water at increasing amounts of CX (Table 1 and Figure S6).

Additionally, in agreement with the 1:1 stoichiometry of the complex, when CX was added to an excess of iodine (iodine:CX, 2:1 molar ratio) in a water solution, the capture of iodine by CX reduced the iodine absorbance by 50% (Figure S7a). As a further confirmation, a reduction by 50% was also found for the absorption of iodine extracted from the water solution by cyclohexane before and after contact with the CX powder (Figure S7b).

Similar results were obtained in phosphate/citrate buffer solution (pH 6). Decreases of 37, 73, 72, and 68% were recorded for the iodine absorption bands at 231 nm, 288 nm, 352 nm, and 453 nm, respectively, after contact with CX powder (iodine:CX, 1:2 molar ratio).

#### 2.2.3. Formation of the Complex by Solid CX and Iodine in Vapor form (Solid–Air Method)

A magnetically stirred powder of CX (50 mg) was placed overnight at 75 °C in a closed container with solid iodine, which sublimates and produces I_2_ vapor. The powder color changed from white to orange and color intensity increased over time. The UV-vis spectrum of the powder dissolved in chloroform showed the typical spectrum of the CX/iodine complex (Figure S8).

**Figure 4:**
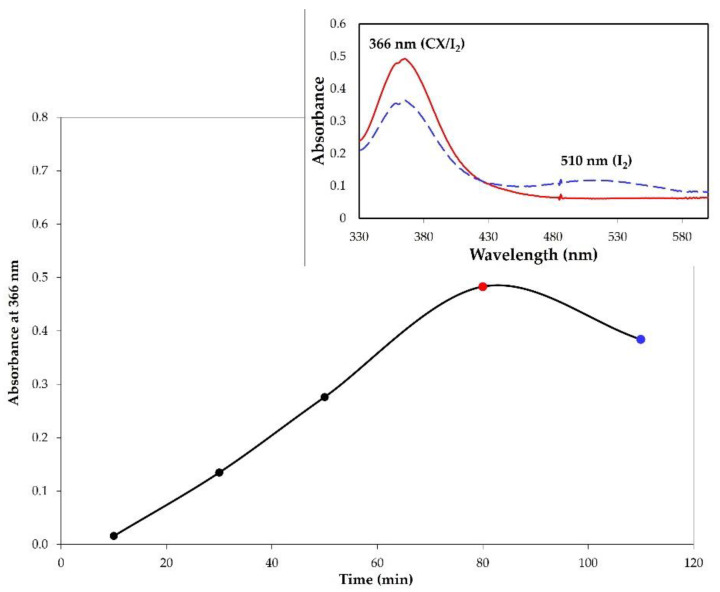
Monitoring of the CX/iodine complex formation by solid–air method. The graphic reports the absorbance values of the complex (λ = 366 nm) as a function of time. Inset: UV-vis spectrum in chloroform of CX/iodine complex after 80 min (solid red line) and 100 min (dashed blue line).

The complex formation was monitored by UV-vis analysis in chloroform at different interval times. The absorbance values at 366 nm were plotted as a function of time (Figure 4). The trend in Figure 4 shows that the complex formation proceeded up to 80 min, after which a reduction in the absorbance was observed. The UV-vis spectrum in chloroform of the sample at 100 min showed, in addition to the band at 366 nm, a band at 510 nm relative to free iodine. This behavior, also observed for other iodophors, indicated that after saturation the adsorption of I_2_ is accompanied with iodine release [9].

**Figure 5:**
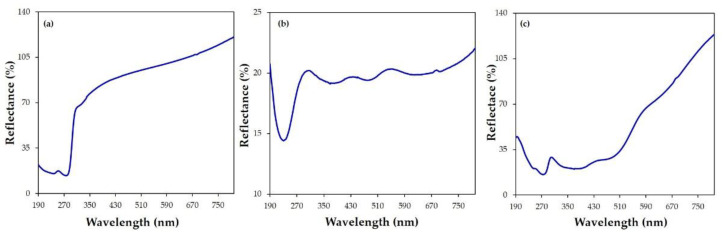
Reflectance (%) spectra of the (**a**) CX, (**b**) iodine, and (**c**) CX/iodine complex powders.

The complexation of I_2_ by CX was corroborated by reflectance (%) spectra. Figure 5 shows the different profile of the reflectance spectra of the solid samples of CX (Figure 5a), iodine (Figure 5b), and the CX/iodine complex (Figure 5c). Typical bands centered at 238 and 274 nm and at 230, 374, 478, and 636 nm were observed for CX and iodine, respectively. The spectrum of the complex (Figure 5c) showed reflectance bands at 243 and 275 nm, ascribable to CX, and 368 and 478 nm, relative to the iodine species.

### 2.3. Stability of the CX/Iodine Inclusion Complex

**Figure 6:**
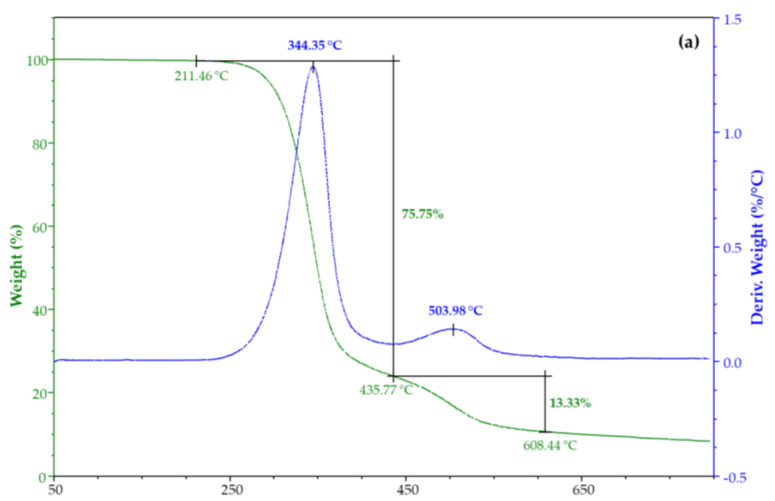

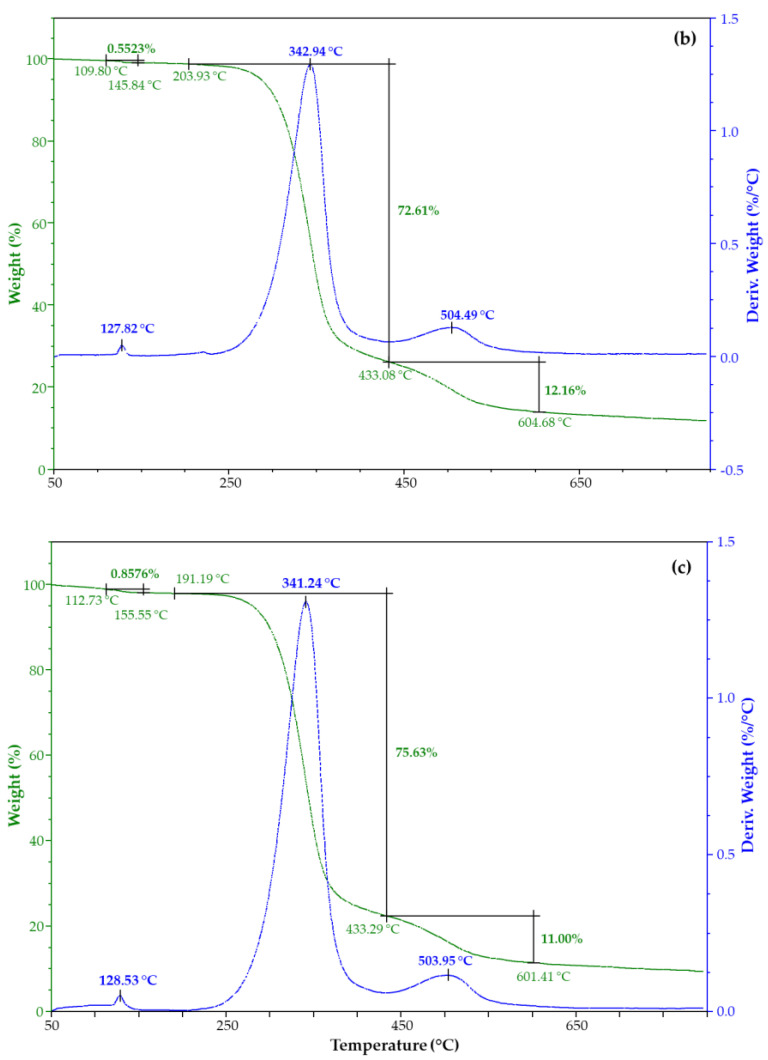
TGA and DTG of (**a**) CX and its complex from iodine, (**b**) water solution, and (**c**) vapor.

The thermal stability of CX and its iodine inclusion complexes was evaluated by TGA. Figure 6 shows their weight loss percentage and derivatives as a function of the temperature. TGA of CX has two degradation steps (Figure 6a), the main being at 344 °C. TGA of both complexes from water solution (Figure 6b) and vapor (Figure 6c) highlighted an additional degradation step at about 128 °C due to iodine (Figure S9).

### 2.4. Calix[4]arene-Polyolefin Systems

Considering the gas permeation mechanism and assuming that the loss of I_2_ is the determining factor for packaging in plastic material, we evaluated different approaches that could repress or reduce the diffusion of I_2_, such as the introduction into the packaging of an additional amount of iodine. The established capacity and stability of CX to complex iodine suggested testing it in comparison with I_2_.

Therefore, CX and its iodine inclusion complex were embedded in polymer matrices (HDPE or PP) via a swelling procedure. Comparative tests were carried out incorporating I_2_ in HDPE or PP. Preliminary tests at room temperature and in pure solvent (Figure S10) were carried out to establish the necessary swelling time.

Consequently, polyolefin film portions were dipped in chloroform solutions of CX, CX/iodine complex, or I_2_ for 24 h at room temperature. After solvent evaporation, surface and bulk changes in the film portions were monitored by contact angle, TGA, and UV-vis.

The embedding of CX in HDPE or PP was performed by dipping the pre-swelled polymer film (chloroform, 24 h) in a chloroform solution of CX (5% and 10% concentration) for 24 h at 25 °C for both HDPE and PP.

**Figure 7:**
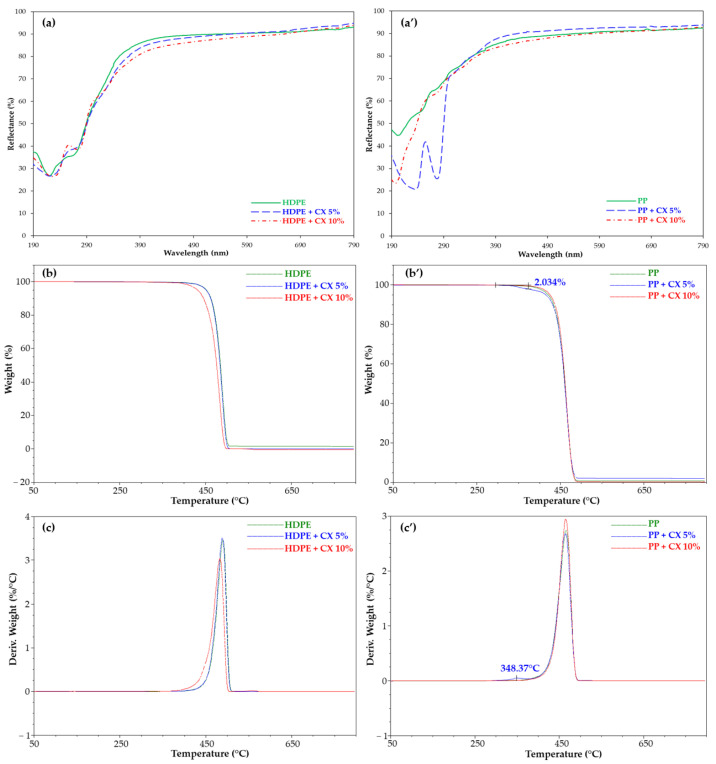
Reflectance (%) spectra, TGA, and DTG of (**a**–**c**) HDPE and (**a’**–**c’**) PP films, empty (green line) and embedded with CX (blue and red lines).

Reflectance (%) spectra (Figure 7a,a’) and TGA analysis (Figure 7b,b’,c,c’) were almost unchanged in HDPE samples, while they clearly evidenced the inclusion of CX in the PP polymer. A band at 230 nm and 276 nm and a peak at 348 °C relative to CX absorption (Figure 7a’) and degradation (Figure 7b’,c’), respectively, were observed. According to TGA, about 2% of the calixarene derivative included in PP was determined for the sample dipped in the 5% CX solution.

The higher affinity of the CX for PP than the HDPE film could be related to the presence of CH_3_ groups in PP that can establish interactions with the CX aromatic rings as well as to the higher swelling index in CHCl_3_ (Figure S10). The unchanged values of the SCA in HDPE embedded with CX at room temperature further confirmed the lower inclusion of CX in the HDPE matrix. Thus, successive tests were focused on the PP polymer matrix, using a 5% CX solution.

Two procedures were carried out to incorporate the CX/iodine complex in PP: a one-step inclusion of the complex and a two-step incorporation, including CX at first and then forming its complex through the exposure of the CX-loaded PP samples to iodine vapor or water solution. A comparative test with iodine-loaded PP samples was carried out through the exposure of the polymer matrices (“empty PP”) to I_2_ vapor or water solution.

**Figure 8:**
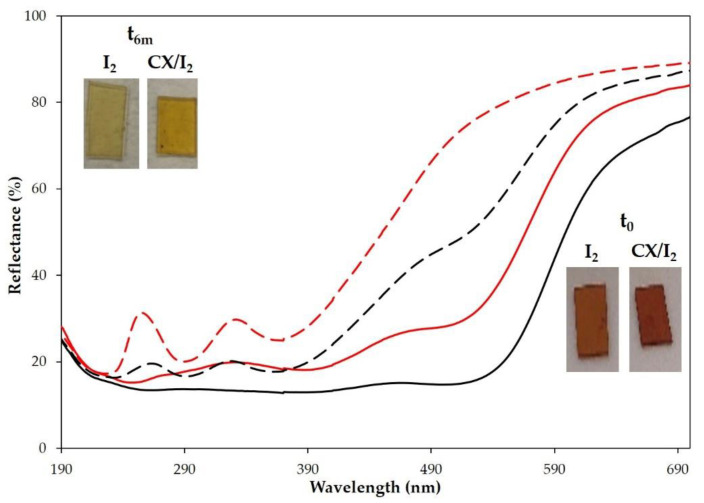
Reflectance (%) spectra of PP polymer films loaded with iodine (red lines) or CX/iodine complex by vapor method (black lines) at t_0_ (full lines) and after 6 months (dashed lines). Insets: pictures of the samples at t_0_ and after 6 months.

For embedding iodine into the CX-loaded or empty PP, the polymer films were dipped into a water solution of iodine (1.18 × 10^−3^ M) or exposed to iodine vapor for 24 h at room temperature. The embedding of iodine in the polymer films was evident with the naked eye from the dark orange coloration and confirmed by reflectance spectra that showed the typical signals of iodine (Figure 8).

It was evident by monitoring the samples over time that the adsorbed iodine was lost from all samples, but a higher amount of I_2_ was retained in the CX-loaded polymers (Figure 8 and insets). Similar results were obtained when the empty films and CX-loaded PP entrapped iodine by treatment with iodine vapors (Figure 8 and insets).

**Figure 9:**
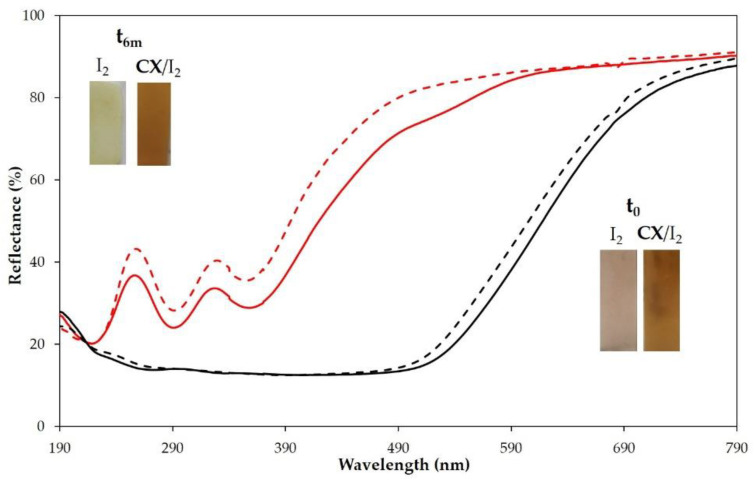
Reflectance (%) spectra of PP polymer films loaded with iodine (red lines) or CX/iodine complex in chloroform solution (black lines) at t_0_ (full lines) and after 6 months (dashed lines). Insets: pictures of the samples at t_0_ and after 6 months.

We also investigated the one-step embedding of the CX/iodine complex into PP films. To these ends, the polymer films, pre-swelled in chloroform, were dipped in a chloroform solution of the CX/iodine complex or iodine as control (7.5 mg/mL chloroform). After solvent evaporation, the polymer films with and without CX showed a different coloration and reflectance spectra (Figure 9, inset). Reflectance spectra confirmed the embedding of iodine and CX/iodine in the PP films. After 6 months, no significant difference in the reflectance spectra of CX/iodide-loaded PP film (Figure 9) was observed, evidencing a good stability of the sample, which is relevant to the evaluation of an eventual barrier effect over time.

**Figure 10:**
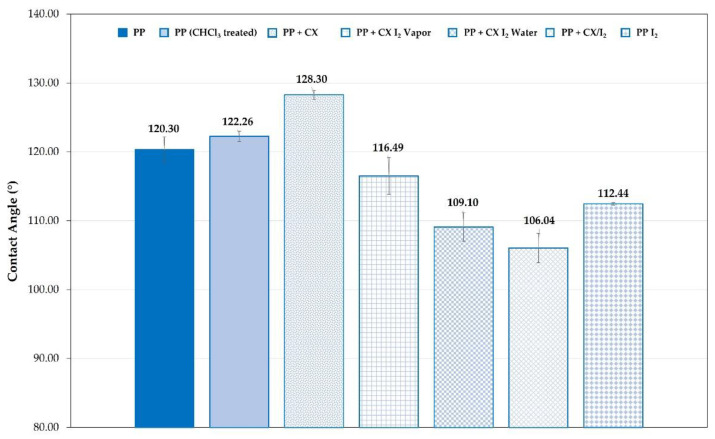
SCA of PP strip samples unmodified and embedded with iodine, CX, or its iodine complex.

Changes in the wettability of the CX-loaded films, consistent with the presence of iodine, were observed as well (Figure 10). In Figure 10, the contact angle values for the embedded PP (Figure 10) samples and unmodified are compared. For PP dipped in the CX solution at 5%, the SCA value is much higher than that of the unmodified polyolefin. This agrees with the lower wettability due to the CX hydrophobicity. The SCA of polyolefin film portions loaded with CX/iodine complex decreased in agreement with the presence of the iodine.

**Figure 11:**
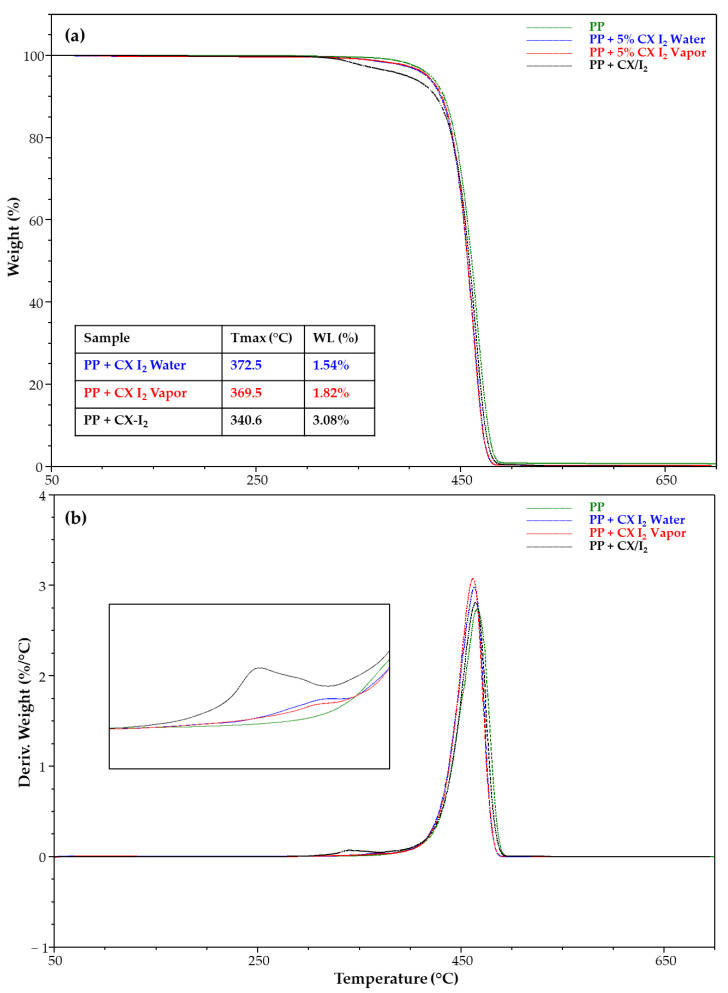
(**a**) TGA and (**b**) DTG of PP strip samples unmodified and embedded with CX iodine complex.

Figure 11 shows the TGA and DTG of the PP strip samples unmodified and embedded with CX and its iodine complex. Noteworthy, TGA highlighted the presence of a degradative step related to the CX/iodine included and allowed the determination of the percentage in the film samples prepared by single- and two-step approaches (Figure 11a). A higher percentage was detected for the PP strip with the CX/iodine complex embedded via a single step.

### 2.5. Influence of Calix[4]arene Derivative-Polypropylene Systems on Leaching of Iodine through Packaging from a PVP-I Buffered Solution

**Figure 12:**
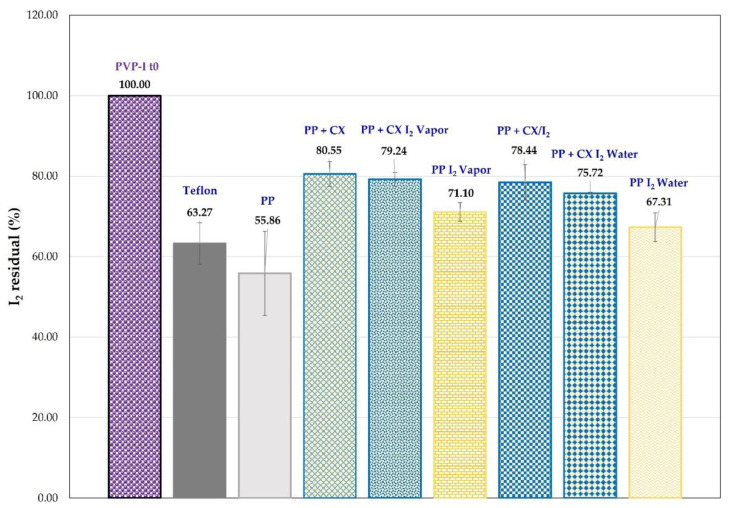
Residual iodine (%) determined in PVP-I buffered solution after 7 days at 40 °C in vials closed with cups of PP samples, unmodified and embedded with CX and its complex and I_2_.

A comparative test was carried out to check the influence of CX-polyolefin systems on the leaching of iodine through packaging from a PVP-I solution (0.3% in buffer, pH = 6) simulating a low-concentration iodine formulation. The I_2_ present in the PVP-I solution was determined by extraction with cyclohexane which, like other organic water-immiscible solvents, removes only the non-ionic iodine (I_2_) and not the anionic iodine species present in PVP-I solutions. The amount of iodine extracted was determined by UV-vis spectroscopy. To simulate the plastic dropper of a container, we replaced the Teflon septum in a gas chromatography vial with PP circular film samples made in the laboratory. To evaluate the barrier effect, comparative tests were carried out with vials containing 0.8 mL of PVP-I phosphate citrate buffered solution (pH 6) and with a septum of circle-shaped PP samples, unmodified and embedded with CX and its iodine complex, as well as I_2_. The samples underwent accelerated testing at 40 °C for up to 7 days. The amount of iodine present in the PVP-I solution, at time = 0 and after 7 days at 40 °C, was determined by cyclohexane extraction. In Figure 12, the residual iodine (%) in the PVP-I buffered solution is reported. The iodine amount in solution decreases for all the samples. However, the residual I_2_ (%) in the PVP-I solution covered by Teflon and the circle-shaped unmodified PP is lower than that found for PP samples embedded with CX and its complexes (PP + CX, PP + CX I_2_ vapor, and PP + CX/I_2_, Figure 12). This behavior highlights higher iodine leaching in the absence of CX. Reasonably, PP + CX absorbs I_2_ vapor from the PVP-I buffered solution and, by reproducing the preparation of the PP + CX I_2_ vapor samples, provides a more valuable barrier effect in comparison with the PP unmodified sample. As expected, the PP samples (PP + I_2_ vapor and PP + I_2_ water, Figure 12) embedded with iodine, without CX, also show a higher residual I_2_ (%) than unmodified PP, rather similar to Teflon.

**Figure 13:**
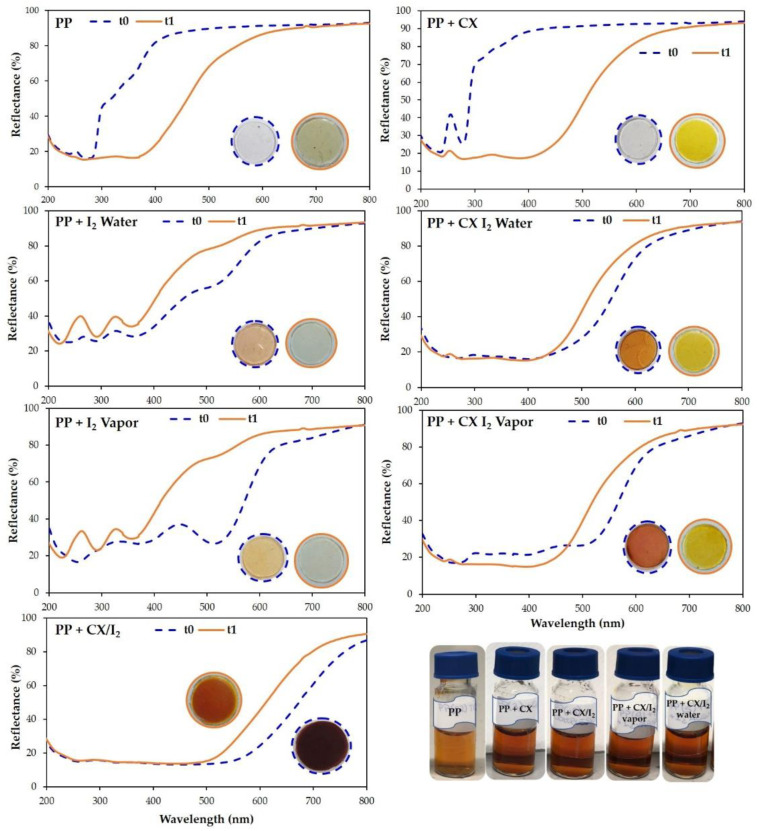
Reflectance (%) spectra and photographic documentation of PP circular film samples unmodified and loaded with iodine or CX/iodine complex at t = 0 (dashed blue line) and after 7 days placed on the cup of a 0.3% PVP-I water solution (solid orange line) at 40 °C.

Reflectance spectra of PP circular film samples, unmodified and loaded with iodine or CX/iodine complex, at t = zero and after 7 days at 40 °C, are reported in Figure 13. The spectra of PP and PP + CX after 7 days clearly highlight a decrease in reflectance (%) in the iodine absorption region, indicative of the absorption of iodine vapor from the PVP-I buffered solution, which is also supported by the yellow coloring of the samples. On the contrary, PP embedded with iodine shows an increase in reflectance and coloring after 7 days at 40 °C due to iodine leaching. Remarkably, no significant variation in reflectance is observed in PP embedded with the CX/iodine complex, since CX successfully retains iodine in its cavity. In agreement with the results revealed in the extraction of residual iodine (Figure 12) from the PVP-I buffered solution, the iodine complexed in the CX provides the more effective barrier effect.

## 3. Materials and Methods

### 3.1. Materials

All reagents and solid iodine were purchased from Sigma-Aldrich Chemical Co., Ltd. (Milan, Italy). PVP-I was obtained from BASF (Milano, Italy). HDPE (PHARMALENE^®^ MP 90 PH, Versalis, Milano, Italy) and PP (Purell HP371P, Lyondellbasell, Milano, Italy) were used for film preparation. Tetra(ethoxycarbonyl-methoxy)-4-tert-butylcalix[4]arene (CX) was prepared as reported in the literature [23].

### 3.2. Preparation of CX/Iodine Complex in Chloroform

CX was added to a chloroform iodine solution (1 × 10^−3^ M) in different molar ratios (from 1:0.5 to 1:3). The mixture was stirred for 15 min at room temperature and analyzed by UV-vis spectroscopy.

### 3.3. Preparation of CX/Iodine Complex from Iodine Water Solution

The water-insoluble powder of CX (200 mg) was added to 200 mL of a 0.3 mg/mL iodine water solution and stirred at room temperature for 24 h. The orange powder of the complex was recovered by filtration, washed with pure water, and dried.

### 3.4. Preparation of CX/Iodine Complex from Iodine Vapors

A magnetically stirred powder of CX was placed in a closed container under iodine vapors at 75 °C. The complex formation was monitored over time.

### 3.5. Preparation of Polyolefin Films

Polyolefin films (average thickness 500 μm) were obtained by a hot press machine PM 20/200 (DGTS srl, Verduggio, Monza Brianza, Italy). Polymer pellets (1.5–2 g) were compressed for 2 min under a pressure of 25–30 bar between two Teflon plates (thickness: 500 μm) containing a spacer (thickness: 500 μm) at 190 °C for HDPE and 210 °C for PP and subsequently water-cooled. Rectangular (0.7 × 4 cm) and circular (0.8 cm) film samples for the different tests were obtained from a cutter. The hot-pressed films were stored at room temperature for at least three weeks before use to reach equilibrium crystallinity.

### 3.6. Embedding of CX/Iodine Complex in HDPE/PP Samples

HDPE/PP samples were pre-swelled in chloroform at room temperature overnight to remove impurities, and then they were dipped in a 5% chloroform solution of the CX/iodine complex prepared by the iodine water solution method and placed in a shaker for 24 h.

### 3.7. Embedding of CX in HDPE/PP Samples

HDPE/PP samples, pre-swelled in chloroform, were dipped in a 5% chloroform solution of CX (5% or 10%). The sample was placed in a shaker overnight and then dried in an oven at 40 °C.

### 3.8. Loading of Iodine in the CX-Embedded PP Samples

The loading of iodine into PP samples embedded with CX was performed by two methods: (A) dipping in a water solution of iodine (0.33 mg/mL) for 24 h and (B) contact with iodine vapor for 24 h at 40 °C.

### 3.9. Nuclear Magnetic Resonance (NMR)

The ^1^H NMR spectrum of CX was acquired on a Bruker Avance 400 spectrometer (400 MHz, CDCl_3_, 297 K). The spectral signals (Figure S1) were consistent with data reported in the literature [23].

### 3.10. UV-Vis Spectroscopy

UV-vis spectra were recorded at room temperature on an Agilent Technologies 8453 UV-Vis spectrophotometer using quartz cuvettes with a path length of 1 cm and Jasco v770 spectrophotometer equipped with an integrating sphere for reflectance measurements.

### 3.11. Thermogravimetric Analysis (TGA)

Thermogravimetric analyses (TGA) were performed using a thermogravimetric apparatus TGA Q500 (TA instruments, New Castle, DE, USA) under a nitrogen atmosphere at a 10 °C/min heating rate, from 50 °C to 800 °C. Sample weight was approximately 5 mg. The weight loss percent and its derivative (DTG) were recorded as a function of temperature.

### 3.12. Contact Angle Measurements

The surface wettability values of samples were measured at room temperature using a contact angle goniometer (OCA15EC, Dataphysics, Filderstadt, Germany). Static contact angle (SCA) values were determined by dropping 2 μL of water from a micro syringe onto the surfaces and analyzing the images taken by the connected video camera with software (SCA 20). To eliminate interference, the samples were previously equilibrated for 30 min at 40 °C and then SCA was measured. At least five measurements were carried out for each sample to ensure repeatability of the experiments.

### 3.13. Determination of Leaching of Iodine through CX-Loaded PP Films

The septum in gas chromatography vials was replaced with PP circular film samples made in the laboratory. A comparative test was carried out to check the influence of CX-polyolefin systems on the leaching of iodine through the packaging from a PVP-I solution (0.3% in phosphate citrate buffer, pH = 6). The I_2_ present in the PVP-I solution was determined by extraction with cyclohexane. The amount of iodine extracted was determined by UV-vis spectroscopy. To evaluate the barrier effect, comparative tests were carried out with vials containing 0.8 mL of PVP-I buffered solution and modified with PP circle-shaped samples, unmodified and embedded with CX (PP + CX) and its iodine complex (PP + CX I_2_ vapor, PP + CX I_2_ water), and I_2_ (PP + I_2_ vapor, PP + I_2_ water). Unmodified PP, PP embedded with CX, or CX-iodine complexes, otherwise known as iodine, and the original septum in Teflon, used for comparison, were used in triplicate. The samples underwent accelerated testing at 40 °C for up to 7 days. The amount of iodine present in the PVP-I solution at time = 0 and after 7 days at 40 °C was determined by UV-vis spectroscopy after extraction with cyclohexane (0.8 mL) at λ = 523 nm by referring to a calibration curve. Weight loss or increase in the PP circular film samples after 7 days was monitored. Reflectance (%) spectra were recorded at time = 0 and after 7 days at 40 °C.

## 4. Conclusions

The capability of a hydrophobic calix[4]arene derivative blocked in a cone conformation to complex iodine from vapor as well as water or chloroform solution was demonstrated by UV-vis spectroscopy. Reducing iodine loss from plastic containers is still a challenge. Thus, to contribute to the research of new approaches to reduce the loss of I_2_ from an iodophor solution, the calix[4]arene/iodine complex was embedded in polyolefin (HDPE and PP) via a swelling procedure. PP showed a higher affinity than HDPE for the CX. The PP films (unmodified and iodine- and calixarene/iodine-loaded) were characterized for surface and bulk changes by contact angle, TGA, and UV-vis diffuse reflectance spectra. A comparative study showed that the films with CX/iodine complex more effectively reduce iodine leaching from a low-concentration PVP-I solution and successfully provide a higher barrier effect.

Overall, preliminary results highlight that calixarene-embedded polyolefins could be promising novel materials to enhance the shelf-life of iodine-based formulations. Further studies are underway for optimizing the embedding of CX and other iodophor calixarene derivatives in polyolefin films. This study paves the way for the development of novel calixarene/polyolefin blends with potential applicability in different fields, including the development of novel materials with antiseptic properties.

## Figures and Tables

**Table 1:** Reduction (%) of the absorbance of the iodine bands in water solution at different iodine:CX molar ratio.

Wavelength (nm)	Absorbance Reduction %		
	1:2 Molar Ratio	1:6 Molar Ratio	1:10 Molar Ratio
288	−36.4%	−61.0%	−93.4%
352	−35.0%	−61.0%	−94.3%
457	−66.3%	−92.5 %	−97.4%

## Data Availability

The data presented in this study are available on request from the corresponding authors.

## Supplementary Materials

The following supporting information can be downloaded at: https://www.mdpi.com/article/10.3390/molecules28041869/s1, Figure S1: ^1^H NMR spectrum of the tetra(ethoxycarbonyl-methoxy)-4-*tert*-butylcalix[4]arene (CX) (400 MHz, CDCl_3_, 297 K); Figure S2: UV-vis spectra of iodine CHCl_3_ solution (1.3 × 10^−3^ M) in the presence of increasing amounts of CX (from 0 to 3.9 × 10^−3^ M); Figure S3: Job’s plot for the determination of the stoichiometry of the CX/iodine complex; Figure S4: Plot of absorbance change versus mole ratio [CX]/[Iodine] at λ = 366 nm; Figure S5: Benesi-Hildebrand plot of absorption data for the CX/iodine complex; Figure S6: UV-vis spectra of iodine in water solution (1.3 × 10^−3^ M) alone and after stirring with increasing amounts of CX powder (iodine:CX 1:2, 1:6, and 1:10 molar ratio); Figure S7: UV-vis spectra of (**a**) iodine in water solution (1.3 × 10^−3^ M) before (blue line) and after stirring with CX powder (iodine:CX, 2:1 molar ratio) (orange line); (**b**) iodine extracted by cyclohexane from the water solution of iodine (1.3 × 10^−3^ M) alone (blue line) and after stirring with CX powder (iodine:CX, 2:1 molar ratio) (orange line); Figure S8: UV-vis spectrum in chloroform of the CX/iodine complex from solid-air method; Figure S9: (**a**) TGA and (**b**) isothermal at 50 °C (60 min) of I_2_ sample; Figure S10: Swelling index in CHCl_3_ vs. time for HDPE and PP strip samples at room temperature.
