# Supplementary material for: Calix[4]arene Derivative for Iodine Capture and Effect on Leaching of Iodine through Packaging
Source: Molecules. 2023 Feb 16;28(4):1869. doi: 10.3390/molecules28041869 (PMC9960756; doi:10.3390/molecules28041869)
Supplement: Supplementary file 1 [file molecules-28-01869-s001.zip › molecules-2228191-supplementary.pdf]

molecules

MDPI

Article

Calix[4]arene Derivative for Iodine Capture and Effect on Leaching of Iodine through Packaging

Loredana Ferreri [1,†], Marco Rapisarda [2,†], Melania Leanza [2], Cristina Munzone [3], Nicola D'Antona [1], Grazia Maria Letizia Consoli [1,*], Paola Rizzarelli [2,*] and Emanuela Teresa Agata Spina [2]

[1] Institute of Biomolecular Chemistry, CNR, Via Paolo Gaifami 18, 95126 Catania, Italy
[2] Institute for Polymers, Composites and Biomaterials, CNR, Via Paolo Gaifami 18, 95126 Catania, Italy
[3] Department of Chemistry, University of Catania, Viale A. Doria 6, 95125 Catania, Italy
* Correspondence: grazia.consoli@icb.cnr.it (G.M.L.C.); paola.rizzarelli@cnr.it (P.R.); Tel.: +39-0957338319 (G.M.L.C.); +39-0957338236 (P.R.)
† These authors contributed equally to this work.

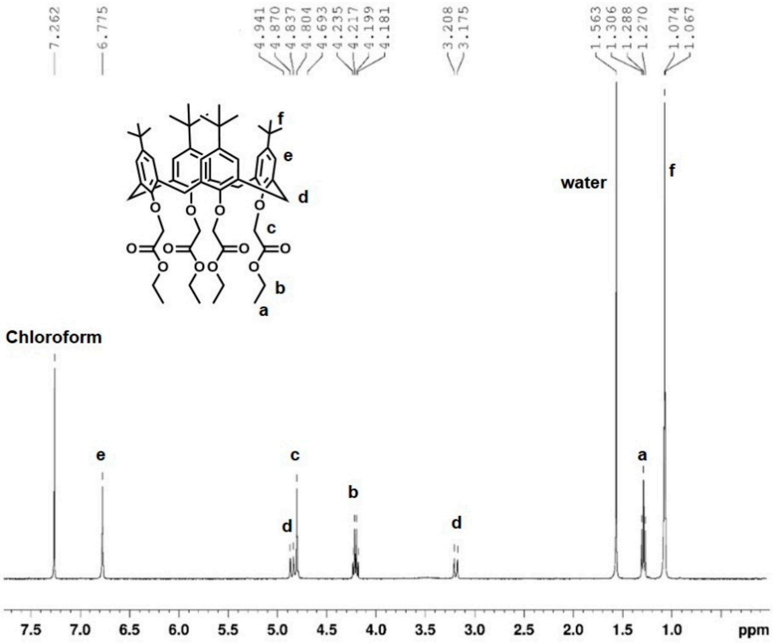

Figure S1. ^1H NMR spectrum of the tetra(ethoxycarbonyl-methoxy)-4-tert-butylcalix[4]arene (CX) (400 MHz, $CDCl_3$, 297 K).

Citation: Ferreri, L.; Rapisarda, M.; Leanza, M.; Munzone, C.; D'Antona, N.; Consoli, G.M.L.; Rizzarelli, P.; Spina, E.T.A. Calix[4]arene Derivative for Iodine Capture and Effect on Leaching of Iodine through Packaging. *Molecules* **2023**, *28*, 1869. https://doi.org/10.3390/molecules28041869

Academic Editor: Paula M. Marcos

Received: 02 February 2023
Revised: 13 February 2023
Accepted: 15 February 2023
Published: 16 February 2023

Copyright: © 2023 by the authors. Licensee MDPI, Basel, Switzerland. This article is an open access article distributed under the terms and conditions of the Creative Commons Attribution (CC BY) license (https://creativecommons.org/licenses/by/4.0/).

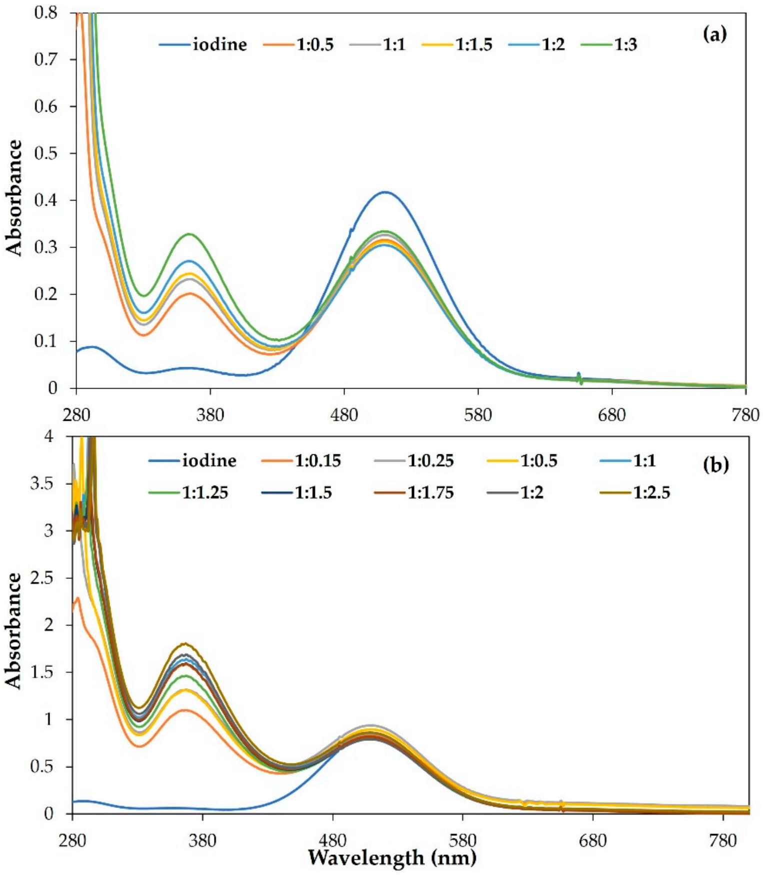

Figure S2. UV-vis spectra of iodine $CHCl_3$ solution (1.3 x 10^{-3} M) in the presence of increasing amounts of CX (from 0 to 3.9 x 10^{-3} M).

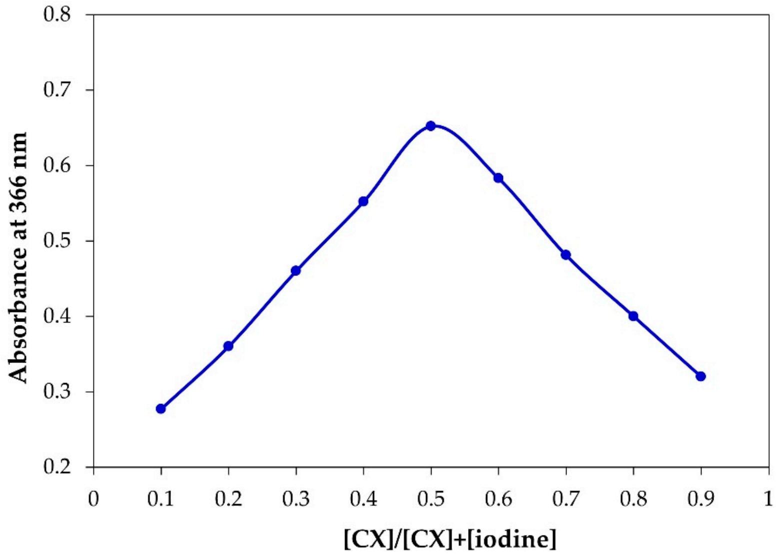

Figure S3. Job's plot for the determination of the stoichiometry of the CX/iodine complex.

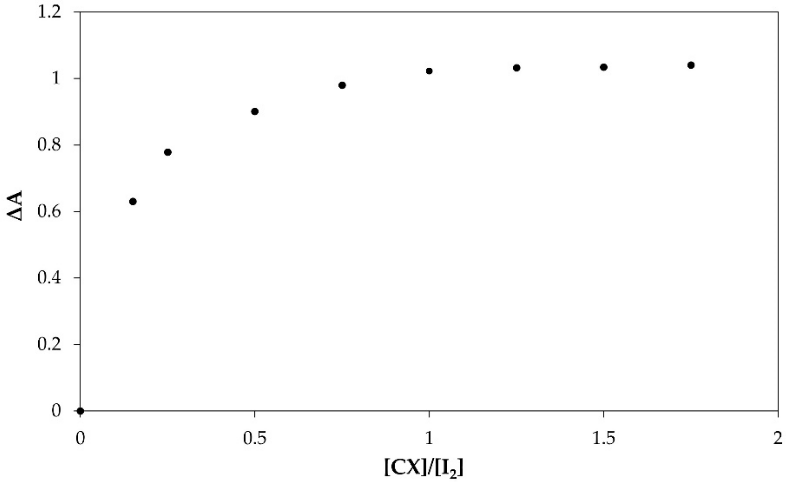

Figure S4. Plot of absorbance change versus mole ratio [CX]/[Iodine] at $\lambda = 366$ nm.

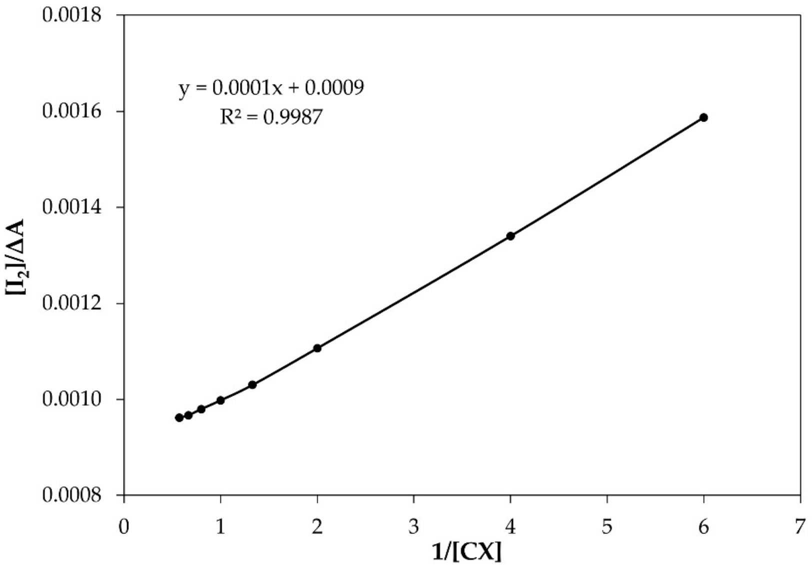

Figure S5. Benesi-Hildebrand plot of absorption data for the CX/iodine complex.

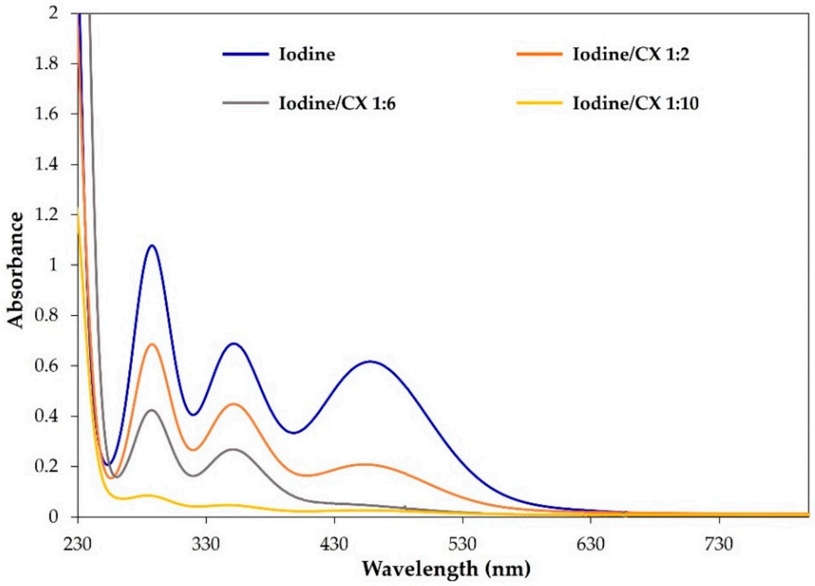

Figure S6. UV-vis spectra of iodine in water solution (1.3×10^{-3} M) alone and after stirring with increasing amounts of CX powder (iodine:CX 1:2, 1:6, and 1:10 molar ratio).

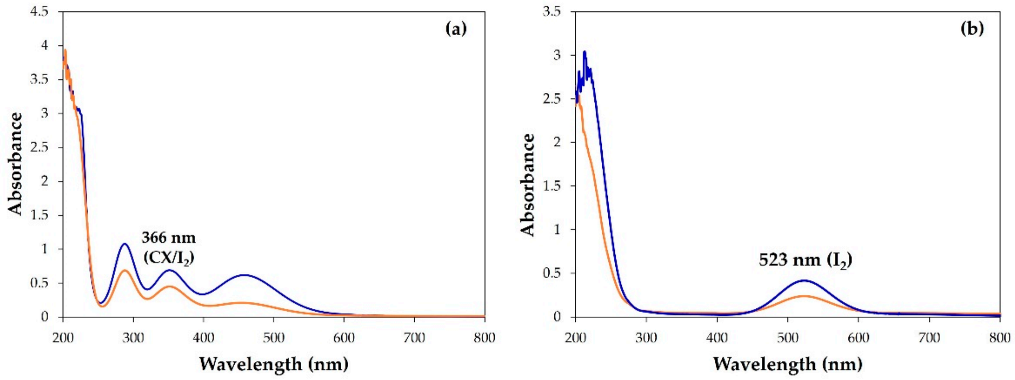

Figure S7. UV-vis spectra of (**a**) iodine in water solution (1.3 x 10^{-3} M) before (blue line) and after stirring with CX powder (iodine:CX, 2:1 molar ratio) (orange line); (**b**) iodine extracted by cyclohexane from the water solution of iodine (1.3 x 10^{-3} M) alone (blue line) and after stirring with CX powder (iodine:CX, 2:1 molar ratio) (orange line).

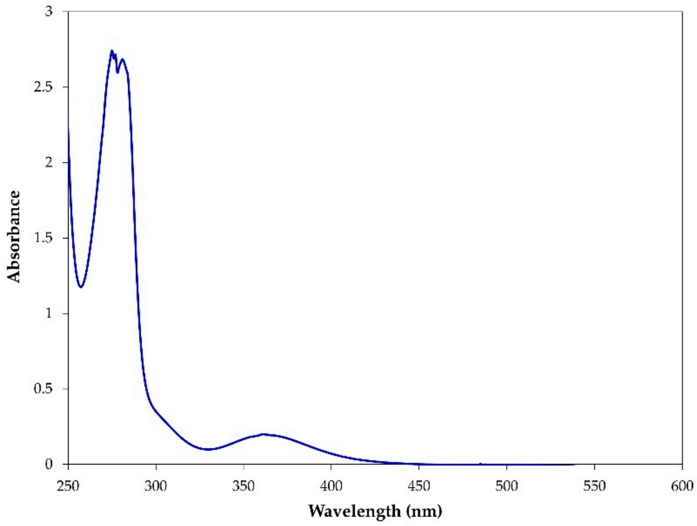

Figure S8. UV-vis spectrum in chloroform of the CX/iodine complex from solid-air method.

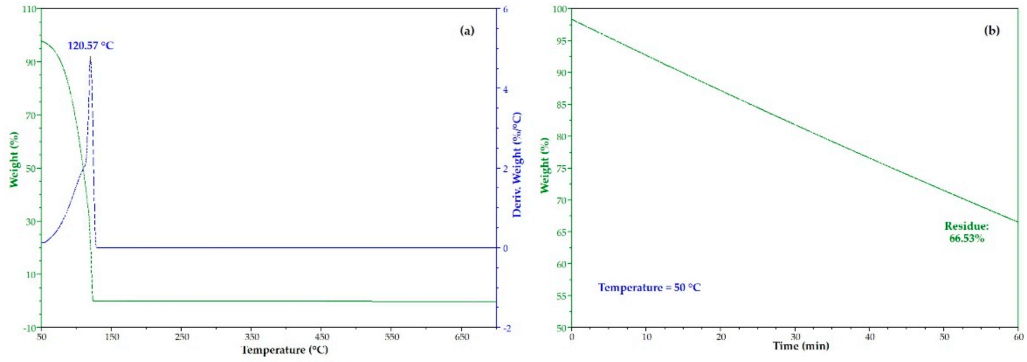

Figure S9. (**a**) TGA and (**b**) isothermal at 50 °C (60 minute) of I_2 sample.

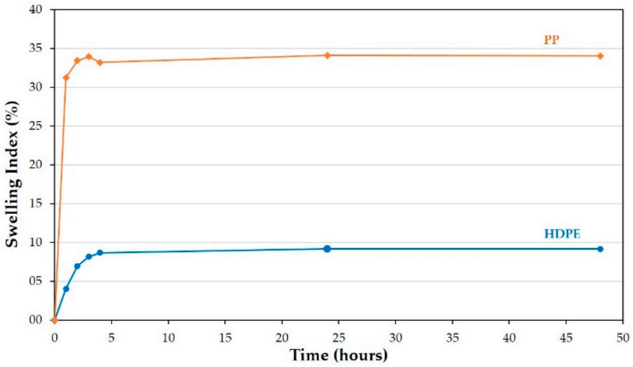

Figure S10. Swelling index in $CHCl_3$ vs time for HDPE and PP strip samples at room temperature.
